# Absence of Siglec-H in MCMV Infection Elevates Interferon Alpha Production but Does Not Enhance Viral Clearance

**DOI:** 10.1371/journal.ppat.1003648

**Published:** 2013-09-26

**Authors:** Franz Puttur, Catharina Arnold-Schrauf, Katharina Lahl, Gulhas Solmaz, Marc Lindenberg, Christian Thomas Mayer, Melanie Gohmert, Maxine Swallow, Christopher van Helt, Heike Schmitt, Lars Nitschke, Bart N. Lambrecht, Roland Lang, Martin Messerle, Tim Sparwasser

**Affiliations:** 1 Institute for Infection Immunology, TWINCORE, Centre for Experimental and Clinical Infection Research: A Joint Venture between the Medical School Hannover (MHH) and the Helmholtz Centre for Infection Research (HZI), Hannover, Germany; 2 Laboratory of Immunology and Vascular Biology, Department of Pathology, Stanford University School of Medicine, Stanford, California, United States of America; 3 Division of Genetics, Department of Biology, University of Erlangen, Erlangen, Germany; 4 Laboratory of Immunoregulation and Mucosal Immunology, Department of Molecular Biomedical Research, VIB, Ghent, Belgium; 5 Institute of Microbiology, Immunology and Hygiene, University of Erlangen, Erlangen, Germany; 6 Institute of Virology, Medical School Hannover (MHH), Hannover, Germany; Oregon Health Sciences University, United States of America

## Abstract

Plasmacytoid dendritic cells (pDCs) express the I-type lectin receptor Siglec-H and produce interferon α (IFNα), a critical anti-viral cytokine during the acute phase of murine cytomegalovirus (MCMV) infection. The ligands and biological functions of Siglec-H still remain incompletely defined *in vivo*. Thus, we generated a novel bacterial artificial chromosome (BAC)-transgenic “pDCre” mouse which expresses Cre recombinase under the control of the Siglec-H promoter. By crossing these mice with a Rosa26 reporter strain, a representative fraction of Siglec-H^+^ pDCs is terminally labeled with red fluorescent protein (RFP). Interestingly, systemic MCMV infection of these mice causes the downregulation of Siglec-H surface expression. This decline occurs in a TLR9- and MyD88-dependent manner. To elucidate the functional role of Siglec-H during MCMV infection, we utilized a novel Siglec-H deficient mouse strain. In the absence of Siglec-H, the low infection rate of pDCs with MCMV remained unchanged, and pDC activation was still intact. Strikingly, Siglec-H deficiency induced a significant increase in serum IFNα levels following systemic MCMV infection. Although Siglec-H modulates anti-viral IFNα production, the control of viral replication was unchanged *in vivo*. The novel mouse models will be valuable to shed further light on pDC biology in future studies.

## Author Summary

Plasmacytoid dendritic cells (pDCs) represent a minor but functionally important subset of dendritic cells. Siglec-H, a surface receptor expressed on these cells, was shown to modulate IFNα production, which in turn could influence anti-viral functions *in vivo*. A potential role for Siglec-H as a pathogen uptake receptor has also been postulated. Yet, the precise *in vivo* function of this molecule in viral replication remained unresolved. In this study, we adopt two novel genetic mouse models to investigate Siglec-H properties and ensuing function *in vivo* during murine cytomegalovirus (MCMV) infection. By using novel reporter mice which harbour permanently labeled Siglec-H^+^ pDCs, we show that pDCs downregulate Siglec-H upon infection. In an additional experimental system, in which pDCs lack Siglec-H function, we demonstrate that this molecule is not important for the regulation of MCMV pathogenicity. In contrast, in the absence of Siglec-H more IFNα was detectable in the serum. Importantly, this *in vivo* increase in IFNα production does not influence viral replication. The biological function of Siglec-H downregulation, also in the context of other infections, requires further investigation.

## Introduction

Dendritic cells (DCs) are a diverse population of professional antigen-presenting cells that exhibit differences in both their developmental origins and their functional properties. They are distributed in different anatomical compartments such as the skin, intestine, lung and lymphoid organs [1], [2] where pathogen access is prevalent. Two prominent murine DC sub-classes exist, conventional DCs (cDCs) and plasmacytoid DCs (pDCs). The latter subset of DCs expresses the I-type lectin receptor Siglec-H. pDCs are implicated in immune tolerance [3]–[8], but are also known to secrete large amounts of type I interferons (IFNs) in response to viral infections [9], [10].

Double stranded DNA viruses like cytomegalovirus (CMV) are sensed by pDCs via the endosomal Toll-like receptor 9 (TLR9) [11] and trigger strong IFNα responses which are critically required for early viral control during MCMV infection [12], [13]. To this end, Zucchini et al. have shown that pDCs are the main early source of intracellular IFNα/β at 30–36 h post MCMV infection [14]. Moreover, Swiecki et al. demonstrated that specific pDC depletion in blood dendritic cell antigen 2 (BDCA2)-diphtheria toxin receptor (DTR) transgenic mice (where ablation of pDCs is mediated by diphtheria toxin) at 36 h post infection (p.i.) resulted in impaired MCMV clearance with unhindered NK cell expansion and function at later timepoints during infection [13]. However, the precise receptors contributing to pDC-mediated anti-viral defence remain incompletely defined.

Amongst the numerous receptors expressed by pDCs, antibody-mediated crosslinking of Siglec-H was shown to negatively influence IFNα production in response to CpG stimulation [15], [16]. This inhibitory regulation of IFNα by Siglec-H was attributed to its association with the ITAM bearing adaptor molecule DAP-12 [16]–[19]. DAP-12 was postulated to recruit inhibitory signalling mediators to dampen TLR-mediated activation. To address this hypothesis, Takagi et al. employed Siglec-H^DTR/DTR^ mice, where an IRES-DTR-EGFP cassette disrupts the Siglec-H open reading frame, harbouring Siglec-H deficient pDCs. HSV-1 infection of these mice induced a strong increase in IFNα levels 6 h p.i. *in vivo* [20]. Additionally, Siglec-H was shown to mediate endocytosis and cross-presentation of antigens suggesting that it may contribute to capturing and delivering of viruses and other pathogens to endosomal TLRs [18]. However, the ligands of Siglec-H remain uncharacterized. Thus, Siglec-H presents as an interesting immunomodulatory molecule pre-dominantly expressed on pDCs, yet its *in vivo* function remains incompletely understood.

To investigate this query, we generated a novel BAC-transgenic Siglec-H reporter mouse (pDCre x RFP) and utilized Siglec-H^−/−^ mice as valuable genetic tools for studying Siglec-H and pDC functions in MCMV infection *in vivo*. Using these novel mouse strains we were able to show downregulation of Siglec-H on pDCs upon MCMV inoculation. Siglec-H is, however, neither required for MCMV infection nor for activation of pDCs. In contrast, Siglec-H deficiency enhances IFNα production without influencing viral clearance.

## Results

### Genetic labeling of pDCs with RFP in pDCre x RFP reporter mice

Since pDCs account for a minor fraction (<0.5%) of immune cells in the spleen [21], we set out to develop a novel transgenic mouse model where Cre recombinase is expressed under the control of the Siglec-H promoter. Transgenic mice were crossed to floxed RFP reporter mice [22], thereby terminally labeling Siglec-H^+^ cells with RFP expression driven from the ubiquitous Rosa26 promoter (Figure S1). The resulting reporter mice are termed pDCre x RFP from here on.

**Figure 1:**
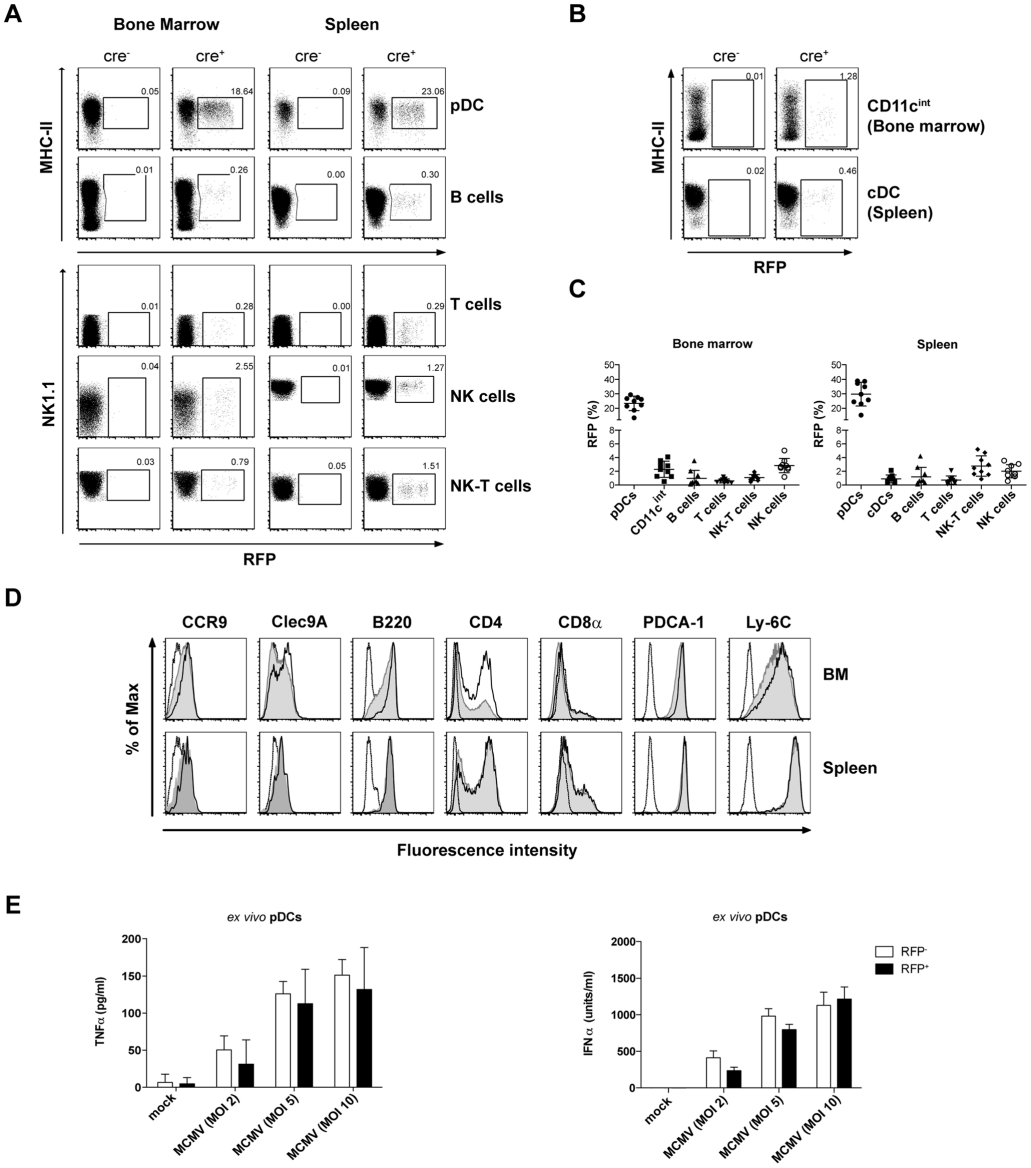
Characterization of the pDCre x RFP reporter line shows terminal targeting of pDCs. (**A, B**) BM and spleen cells from pDCre x RFP mice were stained for Siglec-H, CD11c, MHCII, CD3ε, CD19 and NK1.1 (**A**) Characterization of the reporter expression (RFP) by different cell types: pDCs were gated as Siglec-H^+^, B cells as Siglec-H^−^ CD19^+^ CD3ε^−^ NK1.1^−^, T cells as Siglec-H^−^ CD19^−^ CD3ε^+^ NK1.1^−^, NK-T cells as Siglec-H^−^ CD19^−^ CD3ε^+^ NK1.1^+^, and NK cells as Siglec-H^−^ CD19^−^ CD3ε^−^ CD11c^int^ NK1.1^+^. FACS plots are representative for two individual experiments. (**B**) Characterization of the RFP reporter expression by CD11c^hi^ MHCII^hi^ cDCs in spleen and CD11c^int^ CD19^−^ CD3ε^−^ NK1.1^−^ Siglec-H^−^ cells in BM. (**C**) Quantification of (A, B) showing pooled data from 2 independent experiments using 4–5 mice/group. (**D**) Phenotypic comparison of pDC markers expressed by RFP^−^ and RFP^+^ pDCs from BM (top panel) and spleen (bottom panel). pDCs were gated as Siglec-H^+^ CD11c^int^. Histogram overlays display the isotype controls as dashed line, and the marker expression by RFP^−^ pDCs as grey filled histogram and by RFP^+^ pDCs as bold line. Data shown are from one representative experiment out of two using 4 mice/group. (**E**) Splenic *ex vivo* pDCs were purified by FACS sorting from B16-Flt3L treated pDCre x RFP mice and incubated with the indicated MOIs of MCMV *in vitro*. IFNα/TNFα concentrations were quantified in the supernatants after 24 h incubation by ELISA or cytometric bead assay. Data shown are from one representative experiment out of two using a pool of 3 mice. The differences between RFP^−^ and RFP^+^ pDCs were not significant as calculated by Students t-test. Data displayed in (D, E) are from one out of two individual experiments with similar results. Data are displayed as mean ± SD.

In order to determine the distribution of the reporter expression in pDCre x RFP mice, we characterized cells from bone marrow (BM) and spleen. Gating on individual cell populations revealed that Siglec H^+^ pDCs were targeted most efficiently (Figure 1a, c). Notably, aside of pDCs, we also observed RFP expression by a minor fraction of Siglec-H^−^ cells which consisted of B-, T-, NK-, and NK-T cells from both tested organs as well as splenic cDC and CD11c^int^ BM cells (Figure 1a–c). These results suggest that a small fraction of early lymphoid progenitors actively transcribes the Siglec-H locus and is fate-mapped in pDCre x RFP mice. As previously described, the fluorescence intensity of the RFP reporter differs between cell types and differentiation states and is not a reflection of incomplete recombination [22]. We also found RFP expression by Siglec-H^−^ and Siglec-H^+^ common dendritic cell precursors (CDP) from *in vitro* BM cultures of pDCre x RFP mice (Figure S2a).

Interestingly, we observed RFP expression only by 23±5% (BM) or 30±8% (spleen) of pDCs (Figure 1a, c). Phenotypically and functionally distinct pDC subsets have been reported in humans and mice [23]–[27]. Thus, we performed a pDC surface marker expression analysis including CCR9, Clec9A, B220, CD4, CD8α, PDCA-1, and Ly-6C. When comparing RFP^+^ and RFP^−^ BM pDCs from pDCre x RFP mice, we observed minor alterations in the MFIs of CCR9, Clec9A, B220, PDCA-1 and Ly-6C and more RFP^+^ pDCs expressed CD4 (Figure 1d, top panel). These differences were however not observed in peripheral splenic pDCs with the exception of minor differences in the MFI for CCR9 (Figure 1d, bottom panel). Recently a CD9^+^ pDC subset with potent IFNα production capacities has been described in the BM of mice [27]. We observed comparable targeting of both CD9^+^ and CD9^−^ pDCs in the pDCre x RFP mice (Figure S2b). As we did not identify major phenotypic differences in the surface marker expression of RFP^+^ versus RFP^−^ peripheral pDCs we also wanted to confirm that they possess comparable cytokine production capacities. To this end, we stimulated *ex vivo* purified splenic RFP^+^ and RFP^−^ pDCs from pDCre x RFP mice with different multiplicities of infection (MOI) of MCMV *in vitro* and analyzed their TNFα and IFNα production. As shown in Figure 1e, we did not observe significant differences in the production of the key pro-inflammatory cytokines tested. Hence, given comparable phenotypic and functional characteristics of peripheral RFP^+^ and RFP^−^ pDCs, we report targeting of a representative fraction of pDCs in the pDCre x RFP mice.

### Siglec-H is downregulated upon MCMV infection *in vivo*

**Figure 2:**
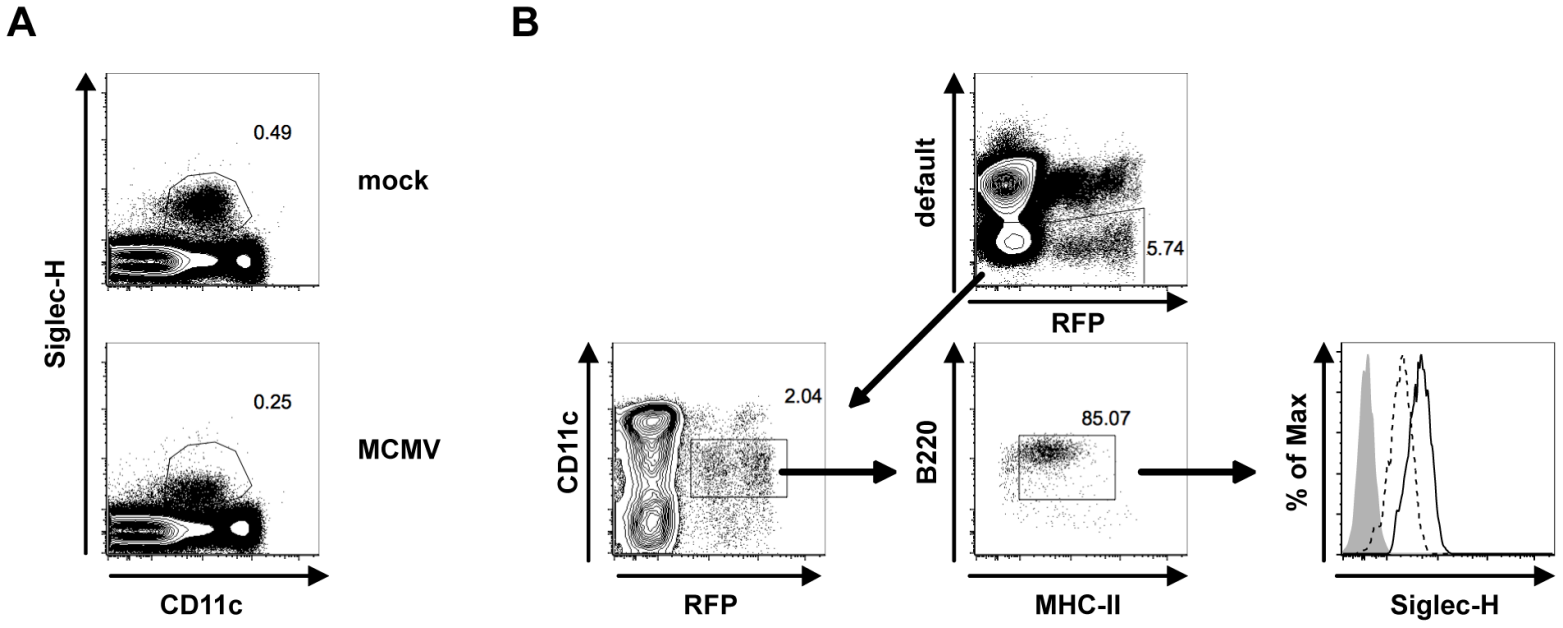
Siglec-H is downregulated upon MCMV infection *in vivo*. pDCre x RFP mice were infected with 5×10^4^ PFU MCMV *in vivo*. (**A**) Representative FACS plots show CD11c versus Siglec-H of live splenocytes at 36 h p.i. (**B**) Gating strategy showing exclusion of B-, T-, NK-T, and NK cells by CD19, CD3ε, NK1.1 in a default channel. RFP^+^ pDCs were gated as CD11c^int^ B220^+^ MHCII^int^ to exclude MHCII^−^ DC precursors. Histogram overlays show RFP^+^ pDCs from mock (bold line) and MCMV infected (dashed line) pDCre x RFP mice at 36 h p.i. Isotype staining is displayed as grey histogram. Data are representative of 2 independent experiments using 4–5 mice with comparable results.

While studying RFP^+^ and RFP^−^ pDCs during MCMV infection, we surprisingly observed downregulation of Siglec-H expression by all pDCs upon MCMV infection *in vivo* (Figure 2a). Thus, defining pDCs by Siglec-H surface expression is hampered. To investigate the extent of Siglec-H downregulation on a per cell basis we used our pDCre x RFP model. The advantage of this model is that pDCs are still fate mapped by RFP expression even after Siglec-H downregulation. Thus, we can accurately gate on the targeted pDC fraction to investigate the consequences of MCMV infection on pDCs. To exclude the non-pDCs targeted in the pDCre x RFP mice a default channel for CD19, CD3ε, and NK1.1 was used. Furthermore, also CD11c^hi^ cDCs and MHCII^−^ CDP precursors [28], [29] were gated out. Thus, in the final gate only RFP^+^ pDCs remained. When comparing RFP^+^ pDCs from mock treated and infected pDCre x RFP mice, we observed Siglec-H downregulation upon infection (Figure 2b) with some cells becoming even Siglec-H^−^. Therefore, Siglec-H surface expression does not faithfully identify pDCs during an ongoing MCMV infection because of its downregulation *in vivo*.

### pDCs downregulate Siglec-H upon MCMV infection in a TLR9- and MyD88-dependent manner

**Figure 3:**
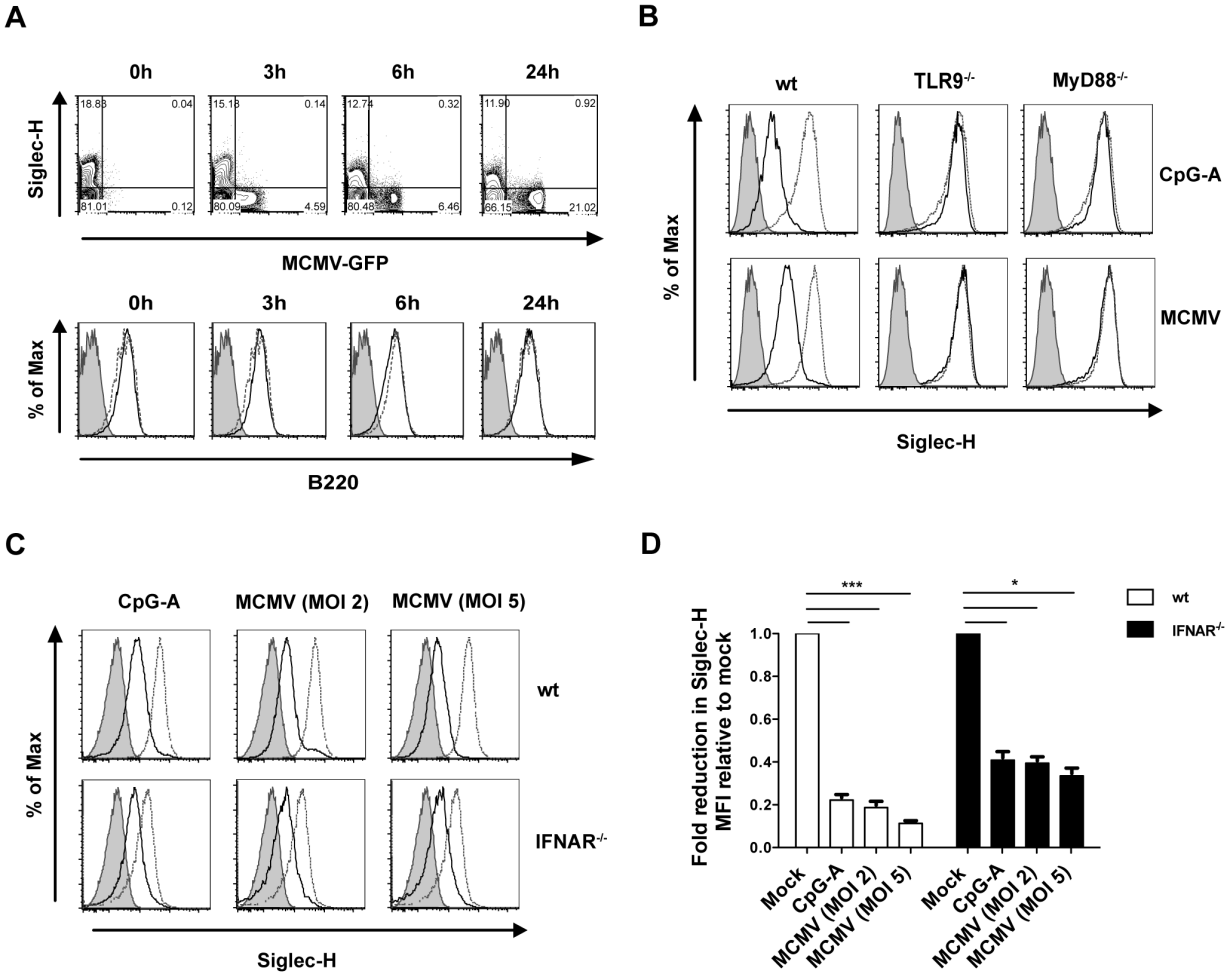
pDCs downregulate Siglec-H upon MCMV infection in a TLR9- and MyD88-dependent manner *in vitro*. Flt3-L derived total BMDCs or CD11c^+^B220^+^ pDCs sorted from BMDC cultures were mock treated with PBS, CpG treated or MCMV infected at MOI 2 unless indicated otherwise. (**A**) Representative FACS plots show Siglec-H expression and MCMV-GFP expression of BMDCs (upper panel). Representative histogram plots display B220 expression on CD11c^+^ Siglec-H^+^ DCs at 0, 3, 6 and 24 h p.i. compared with mock treated controls (lower panel). (**B**) Representative histogram plots display Siglec-H expression of sorted wt, TLR9^−/−^ and MyD88^−/−^ pDCs after stimulation or infection. (**C**) Representative histogram plots display Siglec-H expression of sorted wt and IFNAR^−/−^ pDCs 24 h after infection or stimulation. (**D**) Quantification of the Siglec-H expression from wt and IFNAR^−/−^ pDCs after MCMV infection relative to their respective mock controls. Data are from one of three individual experiments with similar results. *p<0.05, ***p<0.001, Students t-test. (Gray bar = isotype control, gray dashed line = mock treatment and dark bold line = CpG-A treatment or MCMV infection).

As we observed Siglec-H downregulation *in vivo*, we next sought to study the mechanism. For this purpose we infected Flt3-L differentiated bone marrow dendritic cells (BMDCs) with MCMV-GFP and tracked Siglec-H expression *in vitro* over time (Figure 3a). CD11c^+^Siglec-H^−^ DCs express virus encoded GFP starting from 3 h p.i., while both the frequencies of infected DCs and the GFP expression levels progressively increase until 24 h p.i. Interestingly, Siglec-H was downregulated on CD11c^+^ DCs starting from 6 h p.i. (Figure 3a, upper panel), whereas B220 expression remained unaltered at different timepoints p.i. (Figure 3a, lower panel). Interestingly, Siglec-H downmodulation precedes CD86 upregulation (Figure S3b). We next used sorted pDCs from BMDC cultures and found that they also downregulated Siglec-H upon MCMV infection or CpG-A treatment, underlining that accessory cells are dispensable for this effect (Figure 3b). Since pDCs sense MCMV mainly via TLR9 [11], we assessed whether Siglec-H downregulation requires TLR9- and MyD88-dependent signalling. Indeed, Siglec-H downregulation upon CpG-A or MCMV treatment only occurred in wildtype (wt) but not in TLR9^−/−^ or MyD88^−/−^ pDCs (Figure 3b). To test whether the absence of Siglec-H downregulation in TLR9^−/−^ and MyD88^−/−^ pDCs was a consequence of impaired IFNα production, we stimulated IFNAR^−/−^ pDCs. Although we found differences in Siglec-H expression between mock treated wt and IFNAR^−/−^ pDCs (Figure 3c), pDCs still clearly downregulated Siglec-H in the absence of type I IFN signalling upon MCMV infection or CpG-A treatment (Figure 3c, d). Notably, we also observed Siglec-H downregulation upon treatment with the TLR7 agonist R837 (data not shown). Thus, downregulation of Siglec-H is linked to early intracellular activation signals which are dependent on TLR9 and MyD88 signalling with only minor contribution of type I IFN signalling.

### Siglec-H does not modulate pDC infection

**Figure 4:**
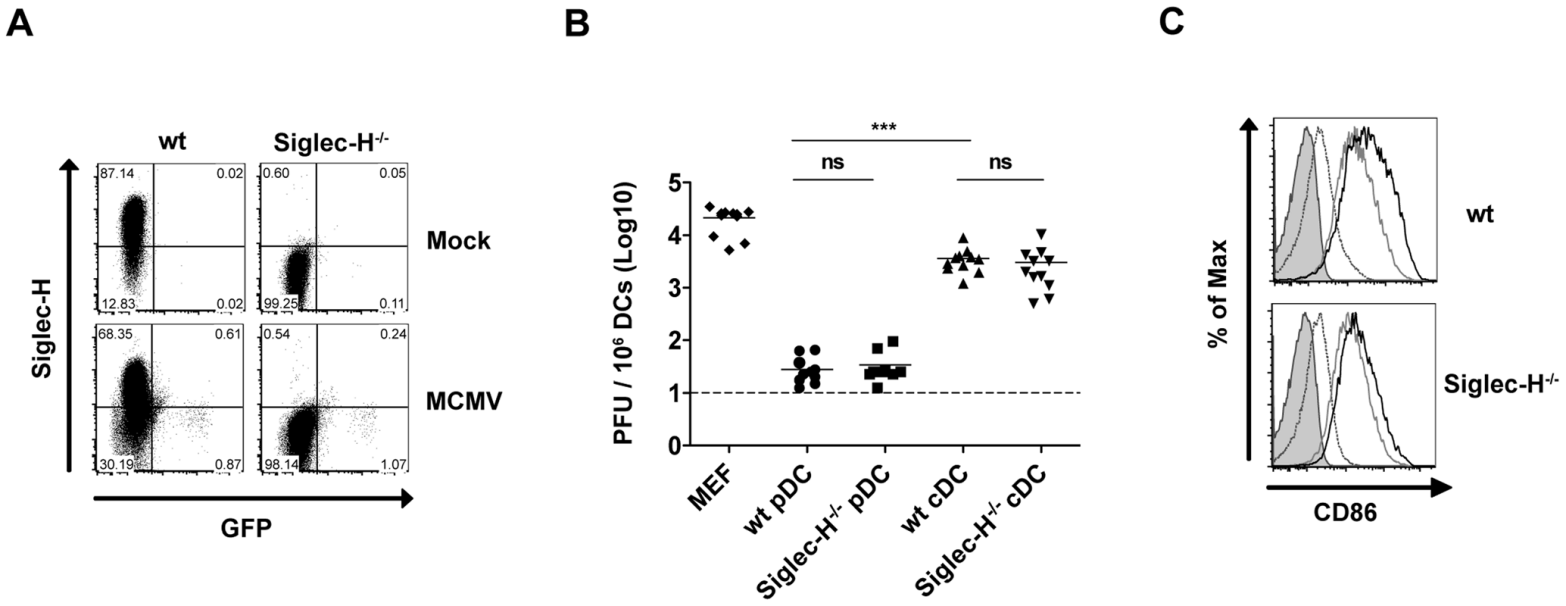
Siglec-H receptor does not play a role in pDC infection. Flt3-L derived CD11c^+^ B220^+^ pDCs and CD11c^+^ B220^−^ cDCs were sorted from BMDC cultures of wt and Siglec-H^−/−^ mice. DCs were mock treated with PBS or MCMV-GFP infected at MOI 2. (**A**) Representative FACS plots show Siglec-H expression and MCMV-GFP expression at 24 h p.i. (**B**) Quantification of the viral titers per 10^6^ DCs or MEFs at 24 h p.i. (**C**) Representative histogram plots of CD86 expression on wt and Siglec-H^−/−^ sorted pDCs (live cell gate) at 24 h p.i. (gray bar = isotype control, gray dashed line = mock treatment, gray solid line = CpG-A treatment, dark bold line = MCMV infection). Data are from one of three individual experiments with similar results. ns = not significant ***p<0.001, Students t-test.

Siglec-H has been postulated as a pathogen uptake receptor [16] and downmodulation of Siglec-H could prevent further infection of pDCs. Thus, we wanted to test for the requirement of Siglec-H for pDC infection/activation. To address this aim, we compared differences in MCMV-GFP infection between sorted Siglec-H^−/−^ and wt pDCs. We observed similiar low frequencies of GFP^+^ wt and Siglec-H^−/−^ pDCs at 24 h p.i. (Figure 4a). Next we quantified the viral titers from MCMV infected sorted pDCs and cDCs from wt and Siglec-H^−/−^ BMDC cultures. Infected cDCs from both groups showed high viral titers. As an additional positive control we included mouse embryonic fibroblasts (MEFs) infected with the same MOI. Wt and Siglec-H^−/−^ pDCs showed basal levels of infection close to the detection limit (Figure 4b) which is consistent with the literature describing pDCs as being resistant to productive infection [12], [30]. Additionally, MCMV infected Siglec-H^−/−^ pDCs showed upregulation of the activation marker CD86 albeit to a slightly lower extent than wt pDCs (Figure 4c). Overall, Siglec-H deficiency on pDCs has no influence on MCMV infection and pDC activation occurs in its absence.

### Siglec-H^−/−^ mice produce significantly more IFNα during the acute phase of MCMV infection

**Figure 5:**
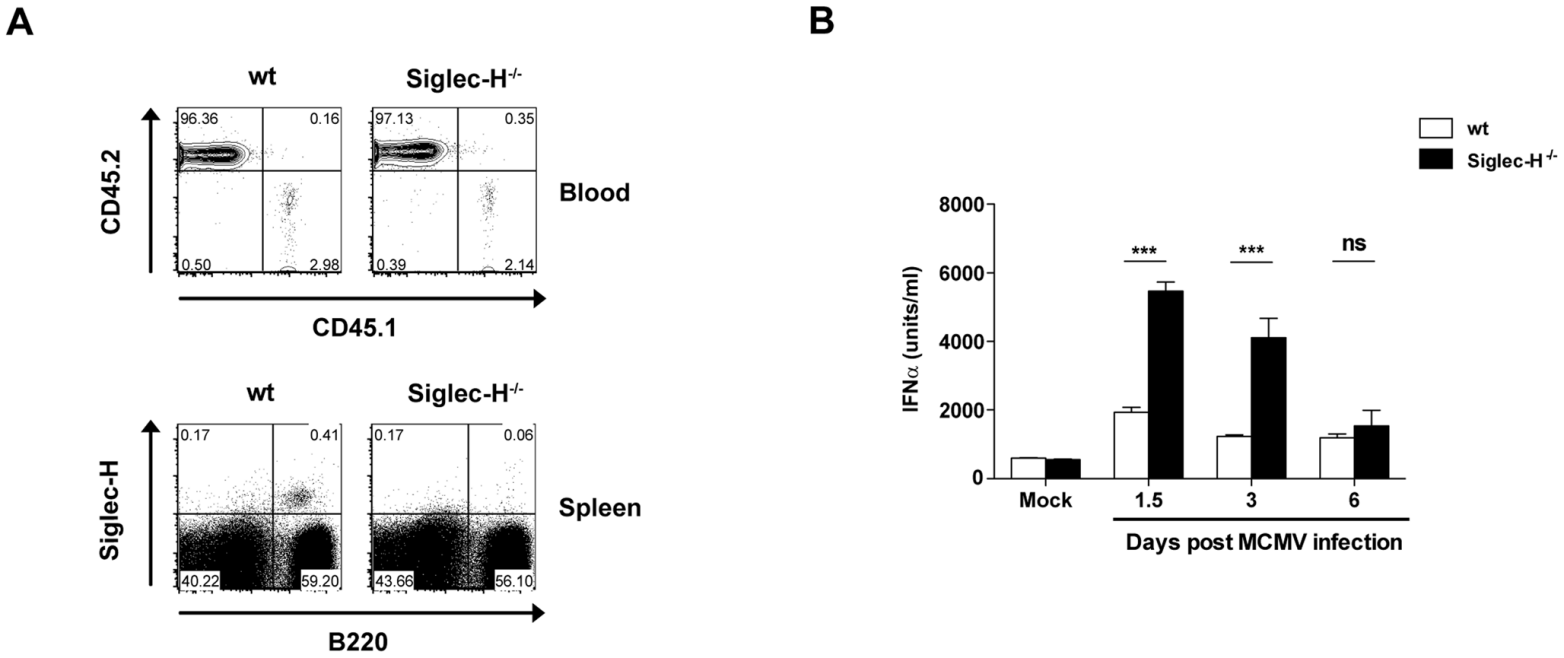
IFNα serum levels are elevated in the absence of Siglec-H upon MCMV infection. Lethally irradiated CD45.1^+^ wt mice were reconstituted with CD45.2^+^ wt or Siglec-H^−/−^ BM followed by infection with 5×10^4^ PFU of wt MCMV. (**A**) FACS plots show efficient donor reconstitution in the blood eight weeks after BM transfer (upper panel). Siglec-H and B220 stainings of splenocytes confirm the lack of Siglec-H expression in wt mice reconstituted with Siglec-H^−/−^ BM (lower panel). (**B**) The kinetics of serum IFNα levels 1.5, 3 and 6 days p.i. compared between Siglec-H^−/−^ and wt mice, n = 4–5 mice/group. Data are from one of two individual experiments with similar results. ns = not significant ***p<0.001, Students t-test.

Siglec-H has a modulatory role in IFN signalling [16]. Thus, we queried whether the absence of Siglec-H would influence anti-viral immunity to MCMV infection *in vivo*. To address this aim, we used BM from wt and the recently described Siglec-H^−/−^ mice [31] to generate chimeric mice and confirmed efficient reconstitution prior to infection (Figure 5a). Subsequently, mice were infected with a low dose of 5×10^4^ plaque forming units (PFU) MCMV and a kinetics of IFNα serum levels was performed. Our results show that at both 1.5 and 3 days p.i. Siglec-H^−/−^ mice had significantly higher IFNα serum levels compared with wt mice (Figure 5b). At day 6 p.i., IFNα levels declined to wt levels in the Siglec-H^−/−^ mice (Figure 5b). Thus, we show that MCMV infected Siglec-H^−/−^ mice produce significantly more IFNα during the acute phase of MCMV infection compared with wt mice.

### MCMV infected Siglec-H^−/−^ and wt mice show similar NK and CD8 T cell responses with equal viral load in primary and secondary infected organs

**Figure 6:**
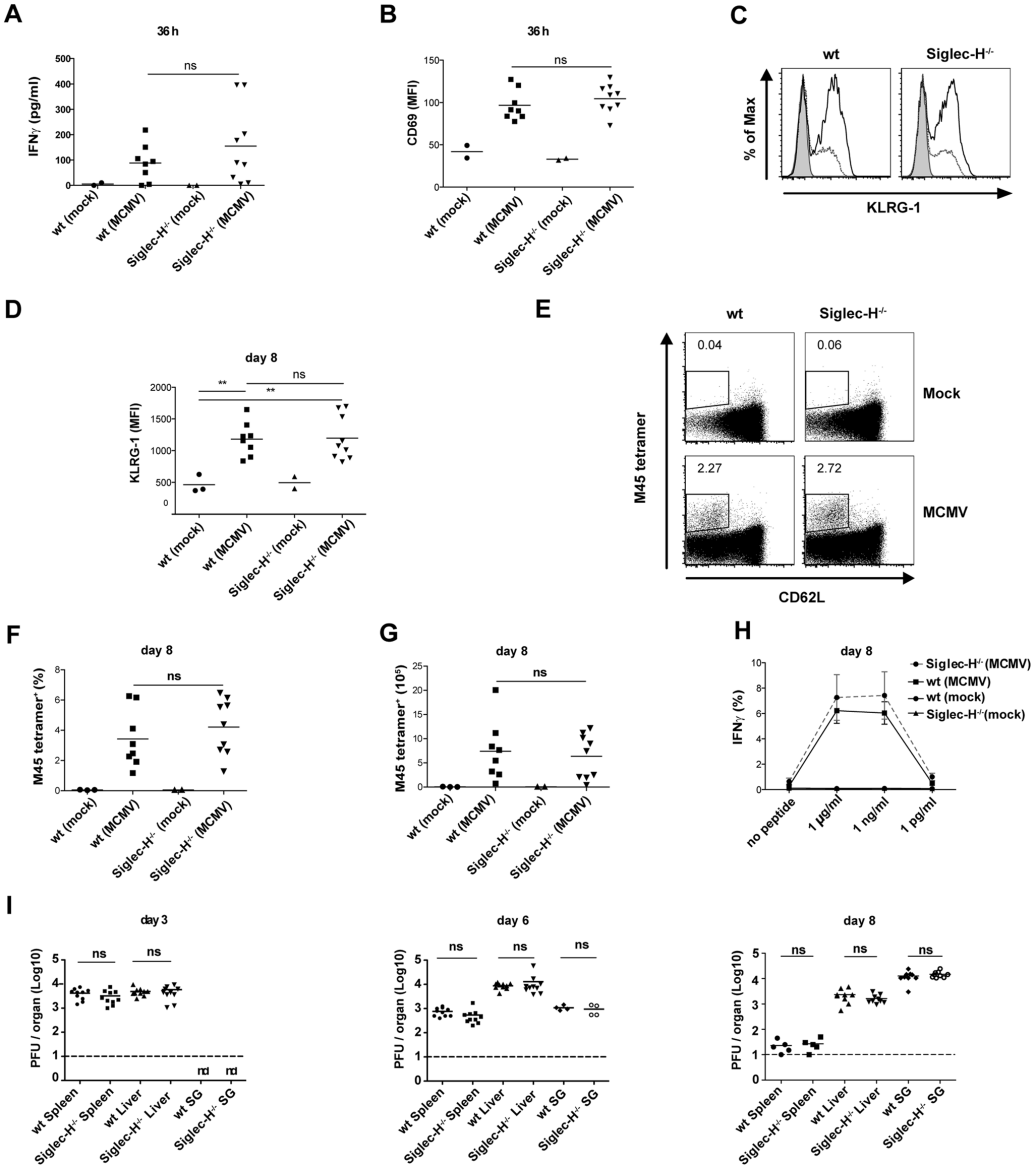
MCMV activates Ly49H^+^ NK cells and expands Ag-specific CD8^+^ T cells from Siglec-H^−/−^ and wt mice with similar viral clearance in the primary and secondary organs. Siglec-H^−/−^ and wt chimeric mice were infected with 5×10^4^ PFU of wt MCMV or mock treated with PBS. (**A, B**) IFNγ serum concentrations were determined at 36 h p.i. and the MFI of CD69 expression on NK1.1^+^ blood NK cells was quantified by flow cytometry. (**C, D**) Representative histogram overlays and quantification of KLRG-1 expression on splenic NK1.1^+^ Ly49H^+^ cells at day 8 p.i. (**E**) Representative FACS plots for H-2D^b^ M45 tetramer staining and CD62L expression among CD8^+^ splenocytes. (**F, G**) Quantification of frequencies and absolute numbers of tetramer^+^ CD8^+^ T cells from the tetramer staining shown in (E). (**H**) Shows frequencies of IFNγ^+^ cells among CD8^+^ T cells after restimulation with the indicated concentrations of H-2D^b^ M45 peptide. (**I**) Viral load was measured in the spleen, liver and salivary glands of MCMV infected Siglec-H^−/−^ and wt mice at day 3, 6 and 8 p.i. Dashed line indicates the limit of detection. Data shown are pooled from 2–3 individual experiments. **p<0.01 Students t-test, ns = not significant, nd = not detectable.

Ablation of pDCs after low dose MCMV infection was shown to enhance the viral load in the spleen, liver and salivary glands of infected mice [13]. However, the functional contribution of Siglec-H in viral clearance remained unknown. As NK cells and CD8^+^ T cells are the major effector cells for MCMV clearance [32], we first tested whether the increase in serum IFNα in infected Siglec-H^−/−^ mice influenced NK cell activation during the early phase of infection. Both serum IFNγ levels and CD69 expression on blood NK cells were comparable between Siglec-H^−/−^ and wt mice (Figure 6a, b). Furthermore we checked for KLRG-1 expression on MCMV-specific Ly49H^+^ NK cells at day 8 p.i. and found a similar activation status when comparing cells from wt and Siglec-H^−/−^ mice (Figure 6c, d).

We next analyzed MCMV-specific CD8^+^ T cells by H-2D^b^ M45 tetramer staining on day 8 p.i. Infected Siglec-H^−/−^ and wt mice showed comparable frequencies (Figure 6e, f) and numbers (Figure 6g) of tetramer^+^CD8^+^ T cells in the spleens of infected mice. Also the IFNγ production by CD8^+^ T cells from both groups was comparable upon restimulation with M45 peptide (Figure 6h).

We finally examined the viral load in the spleen, liver and salivary glands at day 3, 6 and 8 p.i. Importantly, viral clearance in primary and secondary organs showed similar kinetics between the two groups over time (Figure 6i). Infection peaked in the spleen at day 3 and in the liver at day 6 with no significant differences due to Siglec-H deficiency. Also infection in the salivary glands reached the same level on day 6 and 8 p.i. Overall, our data show that absence of Siglec-H does not alter the NK cell activation or expansion of IFNγ^+^ MCMV-specific CD8^+^ T cell generated in response to infection. Additionally, viral clearance in the infected organs remains unchanged irrespective of the presence or absence of Siglec-H.

## Discussion

We have shown that Siglec-H expression is downregulated by pDCs upon CpG-A or MCMV treatment in a MyD88- and TLR9-dependent mechanism. This phenotypic change is observed *in vitro* and *in vivo*. Since a downregulation of Siglec-H expression complicates the identification of pDCs, we generated a novel mouse model for fate mapping Siglec-H expression. In these mice Siglec-H^+^ cells are terminally labeled irrespective of Siglec-H downregulation at a later timepoint. The reporter expression in the pDCre x RFP mice is the highest in pDCs (23–30%) when gating on individual cell populations, as expected. Interestingly, we also observed RFP expression in a minor fraction of B-, T-, NK-, NK-T cells, as well as cDCs and common dendritic cell precursors (CDPs). This might be explained by targeting of a Siglec-H^+^ precursor population as precursor-derived cells would be labeled in our fate mapping approach even in the absence of Siglec-H expression after terminal differentiation. In line with this hypothesis, Siglec-H mRNA expression has been reported recently for B cell progenitors, CDPs as well as common lymphoid progenitors (CLPs) [33]. Furthermore, Satpathy et al. have shown that a fraction of CDPs and particularly a subset of pre-cDCs express Siglec-H at the surface [34]. However, Siglec-H expression by these precursors does not necessarily correlate with pDC differentiation potential and can be transient as a proportion of Zbtb46^+^ Siglec-H^+^ precursors retains cDC differentiation potential [34]. In addition, Swiecki et al. have demonstrated that a fraction of cDCs are targeted in a Siglec-H GFP knock in approach [13]. Supporting these findings, we also report minor targeting of pro- and pre-DCs *in vitro* using Flt3-L cultures of pDCre x RFP BM. Interestingly, a substantial proportion of Siglec-H^−^ precursors is fate mapped, which might indeed indicate that RFP reporter expression precedes Siglec-H surface expression. It will be interesting to test pDCre x RFP mice for possible targeting of CLPs which can give rise to T-, NK-, NK-T and B cells [35]–[38]. To this end, we observed minor RFP reporter expression in early CD3ε^−^ and DN thymocytes (data not shown). It is unlikely that BAC-related positional effects [39] are responsible for the observed expression pattern in non-pDCs as we found similar results in different pDCre founderlines generated from the same BAC (data not shown). Thus, minor cDC, B cell, CDP, and potential CLP targeting observed in the pDCre x RFP mice is likely a reflection of early Siglec-H promoter activity in committed precursors.

Interestingly, not all Siglec-H^+^ pDCs express the reporter in our BAC transgenic model. For both humans and mice, phenotypically and functionally distinct pDC subsets have been described [23]–[27]. However, the expression levels of the pDC surface markers B220, Ly-6C, PDCA-1, CD8α, Clec9A, Siglec-H and CD11c greatly overlapped between RFP^+^ and RFP^−^ pDCs in the spleen. Cisse et al. demonstrated when comparing BMDC cultures from wt and E2-2^−/−^ mice, where pDC differentiation is blocked at an early step, that Ly-6C is upregulated before PDCA-1, B220, and Siglec-H [40]. Furthermore, CCR9 expression is also induced late during pDC development. [24]. Thus, it appeared that RFP^+^ pDCs showed a slightly more mature phenotype in the BM of pDCre x RFP mice. This might indicate that we are preferentially targeting terminally differentiated CCR9^+^ pDCs and fewer Siglec-H^+^ CCR9^−^ B220^low^ pDC precursors [24], [41]. pDCs mainly develop from Lin^−^c-Kit^int/lo^Flt3^+^M-CSFR^+^ CDPs [28], [29]. Recently a progenitor with preferential pDC developmental potential has been identified [42]. Yet, no pDC precursor with a sole pDC differentiation potential has been found. Along this line, it would be interesting to test whether RFP^+^ and RFP^−^ pDCs from the pDCre x RFP mice originate from the same precursor population. Despite the minor phenotypic differences observed in the BM, we did not find a significant disparity in the IFNα or TNFα production upon MCMV infection by peripheral RFP^+^ and RFP^−^ pDCs indicating that we are targeting a representative fraction of cytokine-competent pDCs rather than a distinct pDC subset. We conclude that the pDCre x RFP mice are a suitable model to study cytokine responses by pDCs upon virus challenge.

The injected BAC was chosen to cover approximately 100 kb up- and downstream of the open reading frame to minimize positional effects which are often observed with classical transgenic mice [39], [43]. However, missing regulatory elements required for Siglec-H expression by all pDCs cannot be completely excluded. It should be noted that many helpful transgenic mouse models display a reporter activity that is lower than 100%. ([44], [45] and own unpublished data). This is a commonly observed phenomenon published for different promoters.

We found that Siglec-H downregulation on pDCs is an early response to PAMP recognition *in vitro* and *in vivo*. Productive infection of pDCs with MCMV is, however, low [30], possibly as a consequence of the strong anti-viral response in these cells [12]. Thus, Siglec-H downregulation might be the consequence of pDC activation primarily by means of viral PAMP transfer rather than productive infection as previously shown for other viruses [46], [47]. Because TLR7/9 ligation of pDCs induces a massive type I IFN release, it was important to investigate whether type I IFN signalling alone can induce Siglec-H downregulation. Interestingly, Siglec-H expression by mock treated IFNAR^−/−^ BM-derived pDCs was in general lower as compared with wt pDCs, suggesting a link between IFNAR signalling and Siglec-H expression. Yet, Siglec-H downregulation also occurred in the absence of IFNAR upon MCMV infection. Thus, Siglec-H downregulation is not a direct consequence of type I IFN signalling. Notably, also other soluble factors released by infected wt DCs did not induce Siglec-H downregulation on MyD88^−/−^ pDCs in a transwell experiment (data not shown), arguing for a pDC-intrinsic effect. We conclude that the downregulation of Siglec-H is coupled to early intracellular MyD88-dependent signalling events with minor contributions of the type I IFN signalling pathway.

Siglec-H was previously hypothesized to act as a pathogen receptor although its natural ligands remain enigmatic [16]. However, our data suggest that MCMV infection of pDCs is not affected by the absence of Siglec-H. Moreover, Siglec-H-deficient mice are not protected from MCMV infection. Although Siglec-H seems to be dispensable for infection, this does not exclude that it might participate in virus uptake together with other endocytic receptors with redundant functions.

We show that although Siglec-H is neither required for MCMV replication nor for pDC activation, the absence of this endocytic receptor leads to strongly increased IFNα serum levels in infected mice. It has been previously shown that Siglec-H couples to the adaptor protein DAP12, which negatively regulates type I signalling [19], [48]. DAP12^−/−^ pDCs lack Siglec-H expression and mount higher IFNα levels compared with wt pDCs [19], [48] similar to our findings for the Siglec-H^−/−^ mice. Interestingly, it was demonstrated that DAP12-deficient pDCs also produce increased amounts of IL-12 [19], suggesting that DAP12 may regulate additional DC functions. Whether the absence of Siglec-H or its downmodulation also affects DAP12 expression or function warrants further investigation.

Siglec-H^−/−^ mice showed increased levels of serum IFNα already 36 h p.i. with MCMV. pDCs are the main source of this cytokine at this timepoint [13] and thus the absence of Siglec-H on pDCs has direct consequences for the negative regulation of IFNα signalling by these cells. This finding is consistent with published data from Takagi et al. [20] who demonstrated a similar increase in IFNα serum levels in Siglec-H deficient Siglec-H ^DTR/DTR^ mice in the HSV-1 infection model. In contrast, we did not observe a direct effect of these enhanced cytokine levels on the primary CD8 T cell response or NK cell activation and no differences in viral clearance in spleen, liver or salivary glands during acute infection. This is surprising given that Takagi et al. have shown that in the absence of Siglec-H the HSV-1 specific CD8^+^ T cell response is reduced and viral clearance is diminished [20]. The disparities could be explained by the different mouse or infection model. Alternatively, the wt IFNα serum levels upon MCMV infection might already be saturating.

It is well established that type I IFN promotes anti-viral and immunostimulatory responses [49]–[51]. However, the increased IFNα levels in Siglec-H^−/−^ chimeric mice could also play a dual role and induce negative immune regulation as recently described [52], [53]. Along this line, it would also be interesting to test how Siglec-H^−/−^ mice control reactivation of the virus during chronic infection.

In conclusion, we employed valuable novel mouse models to terminally label a fraction of pDCs and to assess the functional role of Siglec-H *in vivo*. Although Siglec-H is a well defined pDC marker upon steady state conditions, our data suggest that caution should be taken when using Siglec-H as a single marker to identify pDCs upon MCMV infection. This might also apply for other infection models. The mechanisms of TLR-induced Siglec-H downregulation and increased IFNα secretion deserve future investigations.

## Material and Methods

### Ethics statement

All animal experiments were performed in compliance with the German animal protection law (TierSchG BGBl. I S. 1105; 25.05.1998). The mice were housed and handled in accordance with good animal practice as defined by FELASA and the national animal welfare body GV-SOLAS. All animal experiments were approved by the Lower Saxony Committee on the Ethics of Animal Experiments as well as the responsible state office (Lower Saxony State Office of Consumer Protection and Food Safety) under the permit numbers 33.9-42502-04-12/1020 and 33.9-42502-04-09/1785. All surgery was performed after mice were euthanized and all efforts were made to minimize suffering.

### Generation of BAC transgenic pDCre mice

pDCre mice were generated using BAC technology [39], [54] with some modifications. We obtained a BAC encoding the complete mouse *Siglec-H* gene locus (RP24-396N13) from the BACPAC Resources Center at Children's Hospital Oakland Research Institute. As transgene, we created a Cre-IRES-cherry construct containing a 3′ polyA fragment (Figure S1). In contrast to the published overlap PCR strategy [39], we used a strategy based on homologous recombination: 1000 bp long regions homologous to the 5′ end before the ATG of exon 1 and 3′ of exon 1 of Siglec-H were ligated to the transgene construct via PCR-inserted AscI (5′) or PmeI sites (3′). The cherry sequence was a kind gift from Dr. R. Tsien (UC San Diego). The polyA fragment was amplified from the TOPO Tools SV40 pA 3′ element kit (Promega) using primers adding a SpeI site 5′ and a PmeI site 3′. The Siglec-H containing BAC was recombined using the pLD53.SC1 shuttle vector provided by Dr. N. Heintz (The Rockefeller University, New York, NY), gel purified and injected into the pronuclei of fertilized C57BL/6 oocytes. Multiple transgenic pDCre founder lines were established and the pDCre x RFP line with the highest RFP expression of 23±5% in BM- and 30±8% in splenic pDCs was utilized in this paper. Within the RFP^+^ live cell population pDCs constitute 69±8% (BM) and 9±6% (spleen) of total cells. Frequencies are given as mean ± SD. pDCre mice were genotyped by PCR using the following primers: (5′-ttccatggcatgagagaaca-3′) and (5′-agtccagaagcccaaaggat-3′). To analyze for Cre-recombinase activity, the transgenic mice were bred to reporter mice which carry a floxed td-RFP cassette downstream of the ubiquitous Rosa26 promoter [22].

### Generation of Siglec-H^−/−^ chimeric mice

Siglec-H^−/−^ BM was kindly provided by Dr. Lars Nitschke (Erlangen, Germany). Siglec-H^−/−^ mice were generated by the consortium for functional glycomics (http://www.functionalglycomics.org/) [31] and provided by The Scripps Research Institute (TSRI) (La Jolla, CA, USA). The construct consisted of an FRT-flanked selection vector containing a neomycin resistance gene and a single 3′ loxP site was used in the generation of this construct. The selection vector was inserted downstream of exon 2 of the Siglec-H gene and a second loxP site was inserted upstream of exon 1 via homologous recombination. The mice carrying the construct were then crossed with a Cre-recombinase transgenic mouse thereby removing the majority of the coding sequences of Siglec-H including those for the Ig-like domains and alternatively spliced exon 2a.

For generating Siglec-H^−/−^ chimeric mice, CD45.1 mice were obtained from Jackson laboratory, Maine, USA. CD45.1^+^ recipient mice were irradiated with 10 Gy and rested for 24 h after radiation treatment. Irradiated mice were injected intra-venously with 4×10^6^ BM cells from Siglec-H^−/−^ or C57BL/6J control mice (both CD45.2 background). Following injection of BM cells, mice were carefully monitored and reconstitution was carried out for 6–8 weeks. Reconstitution efficiency was examined by staining blood cells for CD45.1 vs. 45.2 after 6–8 weeks. Siglec-H^−/−^ chimeric mice did not show any signs of kidney dysfunction as assessed by serum creatinine measurements.

### Housing

pDCre x RFP reporter and C57BL/6 wildtype (wt) mice (originally obtained from Jackson laboratories) were bred at the animal facility of Twincore (Hannover, Germany) and at the Helmholtz Centre for Infection Research (HZI, Braunschweig, Germany). 8–12 week old mice were used for animal experiments. All animals were housed under specific pathogen-free conditions. Mice were sacrificed by CO_2_ asphyxiation as approved by German animal welfare law. Every effort was made to minimize any sort of suffering to the animals.

### MCMV viral strains

The MCMV laboratory strains used were the BAC-derived wt Smith Strain [55] and the recombinant Smith-based GFP strain [56]. Both strains were kindly provided by Dr. Martin Messerle from the Institute of Virology, Medical School, Hanover, Germany. Virus strains were propagated in mouse embryonic fibroblast (MEF) as described previously [57].

### *In vitro* spin inoculations

1×10^6^ BMDCs/well were seeded in a 24well flat bottom plate. FACS sorted Flt3-L derived pDCs were seeded at 1×10^5^ cells/well in 96well flat bottom plate. DCs were incubated with MCMV Smith-GFP or MCMV Smith (MOI 2), 1 µM CpG-A (ODN 2216) or PBS for 0, 3, 6 and 24 h. Infection of DCs was performed by centrifugation at 1500 rpm for 30 min at 37°C. Following spin infection, DCs were incubated at 37°C, 5% CO_2_ till harvested and used for subsequent stainings.

### *In vitro* stimulations of pDC populations from pDCre x RFP mice

30,000 sorted SiglecH^+^RFP^+^, SiglecH^+^RFP^−^ pDCs were seeded in a 96well U bottom plate and incubated with MCMV (MOI 2 or 5), CpGA-2216 (0.1 µM, 1 µM) or mock infected with PBS. Cells were incubated with appropriate stimuli for 24 h at 37°C, 5% CO_2_. Supernatants were collected and cytokines were quantified as described below.

### *In vivo* systemic infection

Mice were infected intra-peritoneally with 5×10^4^ PFUs of wt MCMV-Smith strain or mock infected with PBS. Mice were carefully monitored over the course of infection and were asymptomatic.

### Quantification of infectious virus

Spleen, liver and salivary glands from sacrificed mice or cell pellets from infected DCs/MEFs (modified protocol from Mathys et al. [56]) were resuspended in MEM medium (Gibco Life Technologies, Darmstadt, Germany). Homogenization was performed by using a handheld homogenizer POLYTRON PT1200 (Fischer-Scientific GmbH, Schwerte, Germany). Organ homogenates were then plated on a confluent layer of murine embryonic fibroblasts (MEFs) and a serial dilution of 10^−1^–10^−6^ was performed for each organ in duplicates. Infections of MEFs were carried out in 24well flat bottom plates for 2 h at 37°C. After 2 h of incubation the organ homogenates were decanted carefully and a layer of methyl cellulose was overlayed to avoid infection by residual free floating virus particles and permit only cell to cell transfer of virus infection. After methyl cellulose overlay, the plates were left at 37°C for 6 days until plaques developed.

### Preparation of single cell suspensions for DC analysis

Spleens were cut into small pieces and digested in RPMI 1640 Glutamax medium (Gibco Life Technologies, Darmstadt, Germany) supplemented with 10% FCS, 5 µM β-mercaptoethanol (Gibco Life Technologies, Darmstadt, Germany), 100 U/ml Penicilin/Streptomycin (Biochrom, Berlin, Germany), 1 mg/ml Collagenase D (Roche diagnostics GmbH, Mannheim, Germany) and 100 µg/ml DNase I (Roche diagnostics GmbH, Mannheim, Germany). Digestion was stopped by addition of 10 mM EDTA (pH 7.2). A single cell solution was prepared and red blood cells were lyzed in RBC lysis buffer (150 mM NH_4_Cl, 10 mM KHCO_3_, 0.1 mM EDTA). The isolated cells were counted by trypan blue exclusion and adjusted to the same cell number for FACS staining.

### Preparation of BM and *in vitro* generation of BMDCs

BM cells were isolated from femurs and tibiae. *In vitro* generation of fms-like tyrosine kinase 3 ligand (Flt3-L) driven DCs from BM has been described previously [58]. 15×10^6^ BM cells were seeded in 10 ml supplemented RPMI 1640 Glutamax medium (Gibco Life Technologies, Darmstadt, Germany) containing Flt3-L (self-made from CHO Flt3-L FLAG cells [58]). Cells were cultured for 9 days at 37°C, 5% CO_2_ before stimulation experiments were performed. Flt3-L producing CHO cells were generated by Dr. Nicos Nicola and kindly provided by Dr. Karen Murphy, WEHI, Melbourne, Australia.

### Stainings for flow cytometric analysis

Cells were washed in PBS and stained with the live/dead fixable aqua dead cell stain kit (Invitrogen, Life Technologies GmbH, Darmstadt, Germany) to exclude dead cells. Cells were washed with PBS and incubated in FACS buffer (0.25% BSA/2 mM EDTA in PBS) containing Fc-block (CD16/32, 2.4G2) for 10 min on ice. Cells were stained with fluorescently labeled antibodies (Ab) for cell surface markers. Stainings were performed for 20 to 30 min on ice. Cells were fixed with 2% PFA in PBS for 20 min on ice. Where indicated, cells were washed with permeabilisation buffer (0.25% BSA/2 mM EDTA/0.5% saponin in PBS) and stained intracellularly for IFNγ 20 min on ice. Samples were stored at +4°C, acquired on a LSRII flow cytometer (BD Bioscience GmbH, Heidelberg, Germany), and analyzed using FlowJo software (Tree Star, Inc. Ashland, USA). Single stains and fluorescence minus one controls were used for accurate gating and compensation. Non-specific binding was estimated by isotype controls and cellular aggregates were excluded by SSC-W.

### Antibodies

All antibodies were purchased from eBioscience (Frankfurt, Germany) if not stated otherwise.

The following fluorochrome labeled anti-mouse antibodies were used:

For pDCs after *in vitro* infection: B220 (RA3-6B2), CD11c (N418), CD86 (GL1), MHCII (AF6-120.1) and Siglec-H (440c). For pDC markers: CD11c (N418), B220 (RA3-6B2), PDCA-1 (eBio927), Ly-6C (HK1.4), CD9 (MZ3), CCR9 (242503, R&D), Clec9A (7H11, Miltenyi), Siglec-H (440c), CD4 (GK1.5) and CD8α (53–6.7). For characterization stainings of spleen and BM from pDCre x RFP mice: NK1.1 (PK136), CD3ε (145-2C11), CD19 (eBio1D3), CD11c (N418), Siglec-H (440c), MHCII (M5/114.15.2).

For blood NK cells: NK 1.1 (PK136), Ly49H (3D10), CD69 (H1.2F3), and CD3ε (145-2C11). For peptide restimulated splenocytes: CD8α (53–6.7), CD62L (MEL-14), CD44 (IM7), KLRG1 (2F1) extracellularly and IFNγ (XMG1.2) intracellularly. H-2D^b^ M45 tetramer (PE-conjugated, a kind gift from Dr. Luka Cicin-Sain) stainings were performed in a 96well U bottom plate at 2×10^6^ splenocytes/well after surface staining for 1 h on ice.

### Cell sorting

Cell sorting was carried out at the Cell Sorting Core facility of the Hannover Medical School using the FACSAria (BD) or XDP MoFlow (Beckman Coulter) FACS sorting machines. pDCs were sorted as CD11c^int^ Siglec-H^+^ NK1.1^−^ CD19^−^ CD3ε^−^ after enrichment from spleens of B16-Flt3L treated pDCre x RFP mice using Optiprep gradient (Axis Shield, Oslo, Norway). Cells were sorted with a 100 µm nozzle, collected in filtered FCS and kept on ice till use. The purity of the sorted cells was determined for each sample and was >95–98%.

### T cell restimulation assay

T cell restimulation assay was performed in a 96well U bottom plate. 2×10^6^ splenocytes from mock treated and day 8 MCMV infected wiltype and Siglec-H^−/−^ chimeric mice were incubated in the presence of 3 µg/ml brefeldin A (eBioscience, Frankfurt, Germany) and M45 H-2Db peptide (HGIRNASFI, Pro-Immune, Oxford) at 1 µg/ml, 1 ng/ml and 1 pg/ml final concentration or without peptide at 37 C, 5% CO_2_ for 4 h.

### Cytokine quantification

Supernatants from stimulated pDCs and serum samples from infected mice were used to determine cytokine concentration by ELISA or by FlowCytomix bead assay. For the IFNα ELISA, maxisorp plates (Nunc, Denmark) were coated with anti-mouse IFNα (clone: RMMA-1) and blocked using 1% BSA in PBS. Samples were incubated overnight at 4°C. A poly-clonal anti-IFNα antibody from rabbit (both IFNα antibodies from PBL Interferon Source, NJ, USA) and a HRP conjugated anti-rabbit IgG (H+L) antibody (Dianova, Hamburg, Germany) were used as secondary reagent. HRP activity was detected by BD OptEIA solutionA/B (BD Bioscience GmbH, Heidelberg, Germany) and quantified at 570/450 nm. TNFα and IFNγ were quantified by a FlowCytomix bead assay (eBioscience, Frankfurt, Germany).

### Statistics

The Students t-test was used to calculate the statistically significant differences between samples. A value for p<0.05 was considered significant indicated by an asterisk sign: (*) for P<0.05, (**) for P<0.01 and (***) for P<0.001.

## Supporting Information

Figure S1 **Construct design of the pDCre mouse by targeting the Siglec-H promoter with BAC gene technology.** Shown here is the design of the Siglec-H BAC construct displaying the recombination site within the Siglec-H exon 1 locus. Mice carrying this construct expressed Cre recombinase under the influence of the Siglec-H promoter and were further bred to floxed RFP reporter utilizing the ubiquitous Rosa 26 promoter to generate the pDCre x RFP reporter mice.
(TIF)

Figure S2 **Reporter expression in early dendritic cell precursors and CD9^+^ pDC.** (**A**) Shows an *in vitro* CDP differentiation assay from CFSE labeled BM from pDCre x RFP mice or littermates analyzed on day 3 of Flt3-L culture based on a protocol from Naik et al. [28]. Ly-6G, MHCII, CD19, and CD127 were excluded in a default channel. Pro-DCs were gated as CFSE^low^ CD11c^−^ and pre-DCs as CFSE^low^ CD11c^+^. Representative FACS plots showing Siglec-H (or isotype control) staining versus RFP reporter expression from one out of two independent experiments. (**B**) CD9 expression by BM pDCs from pDCre x RFP mice or littermates gated as Siglec-H^+^ CD11c^int^ from one out of two independent experiments.
(TIF)

Figure S3 **Co-expression of CD86 versus Siglec-H on BMDCs at 0, 3, 6 and 24 h p.i.** Flt3-L derived mixed BMDCs were MCMV-GFP infected at MOI 2. Cells were gated on live CD11c^+^ GFP^−^ and CD11c^+^ GFP^+^ DCs. (**A**) Gating strategy. (**B**) Co-expression of Siglec-H versus CD86 expression. Results are representative for 3 independent experiments.
(TIF)
