# Supplementary figures and images for: Absence of Siglec-H in MCMV Infection Elevates Interferon Alpha Production but Does Not Enhance Viral Clearance
Source: PLoS Pathog. 2013 Sep 26;9(9):e1003648. doi: 10.1371/journal.ppat.1003648 (PMC3784486; doi:10.1371/journal.ppat.1003648)

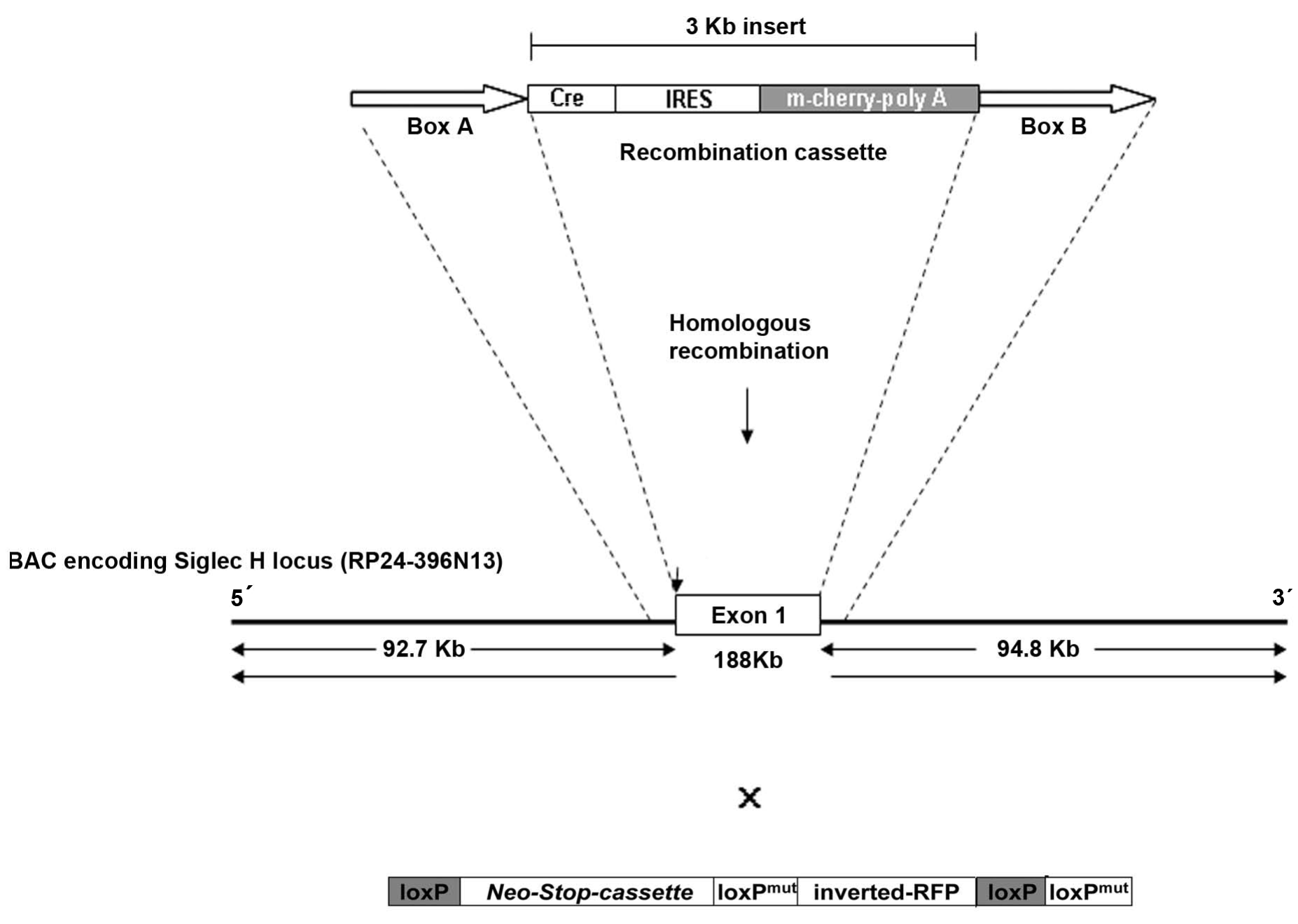

Supplement: Figure S1 — Construct design of the pDCre mouse by targeting the Siglec-H promoter with BAC gene technology. Shown here is the design of the Siglec-H BAC construct displaying the recombination site within the Siglec-H exon 1 locus. Mice carrying this construct expressed Cre recombinase under the influence of the Siglec-H promoter and were further bred to floxed RFP reporter utilizing the ubiquitous Rosa 26 promoter to generate the pDCre x RFP reporter mice. (TIF) [file ppat.1003648.s001.tif]

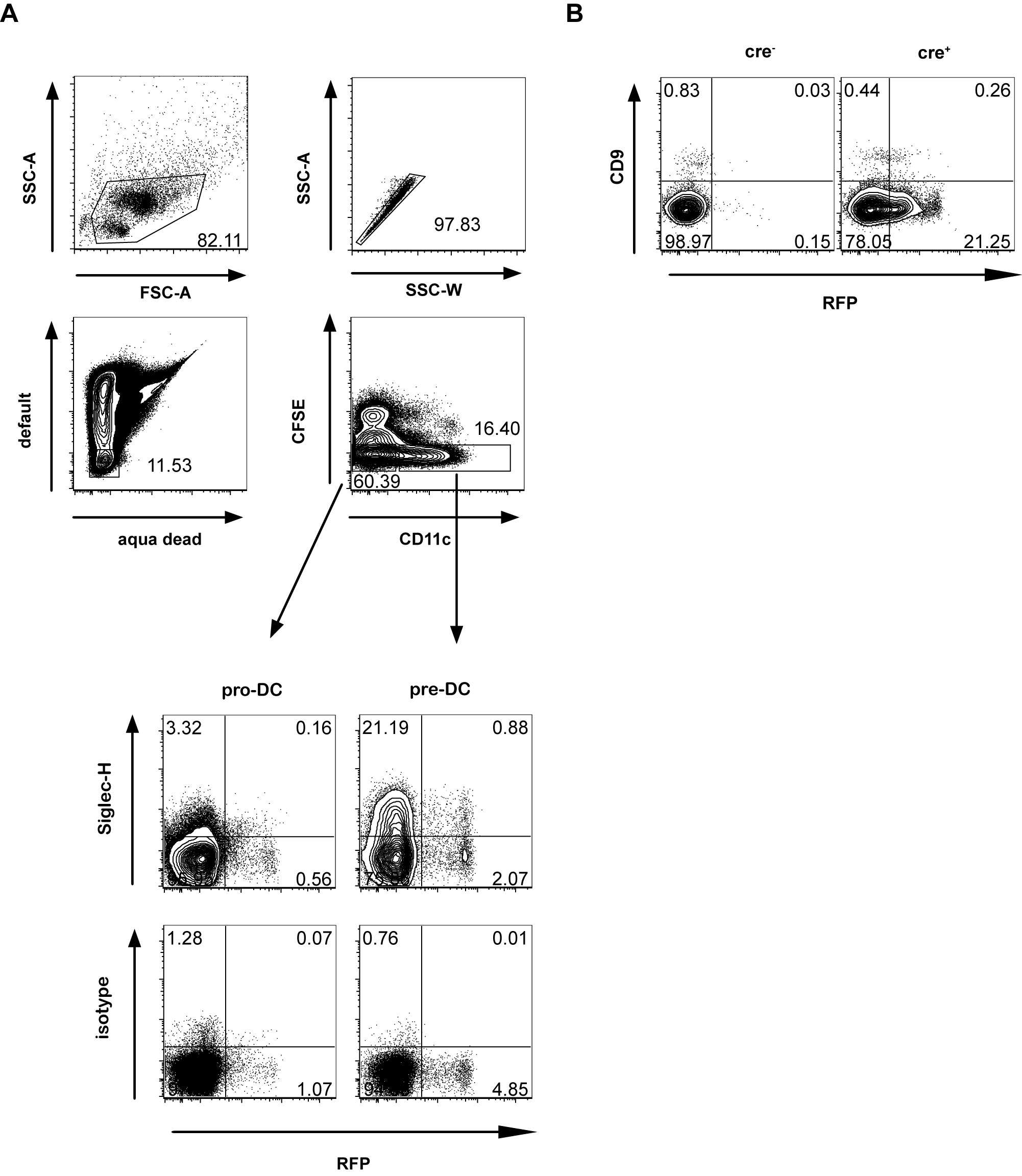

Supplement: Figure S2 — Reporter expression in early dendritic cell precursors and CD9+ pDC. (A) Shows an in vitro CDP differentiation assay from CFSE labeled BM from pDCre x RFP mice or littermates analyzed on day 3 of Flt3-L culture based on a protocol from Naik et al. [28]. Ly-6G, MHCII, CD19, and CD127 were excluded in a default channel. Pro-DCs were gated as CFSElow CD11c− and pre-DCs as CFSElow CD11c+. Representative FACS plots showing Siglec-H (or isotype control) staining versus RFP reporter expression from one out of two independent experiments. (B) CD9 expression by BM pDCs from pDCre x RFP mice or littermates gated as Siglec-H+ CD11cint from one out of two independent experiments. (TIF) [file ppat.1003648.s002.tif]

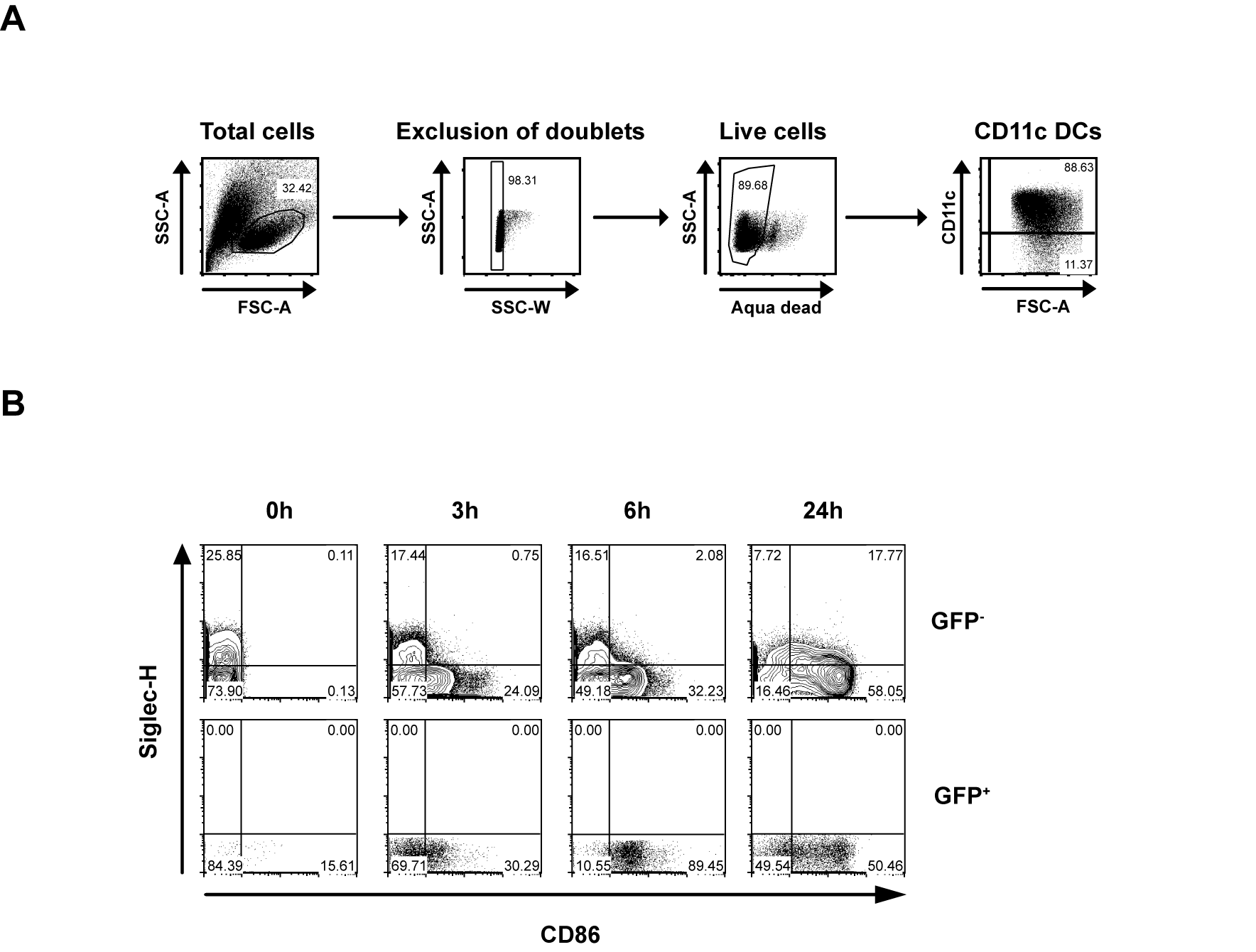

Supplement: Figure S3 — Co-expression of CD86 versus Siglec-H on BMDCs at 0, 3, 6 and 24 h p.i. Flt3-L derived mixed BMDCs were MCMV-GFP infected at MOI 2. Cells were gated on live CD11c+ GFP− and CD11c+ GFP+ DCs. (A) Gating strategy. (B) Co-expression of Siglec-H versus CD86 expression. Results are representative for 3 independent experiments. (TIF) [file ppat.1003648.s003.tif]
